# Tumor heterogeneity of pancreas head cancer assessed by CT texture analysis: association with survival outcomes after curative resection

**DOI:** 10.1038/s41598-018-25627-x

**Published:** 2018-05-08

**Authors:** Gabin Yun, Young Hoon Kim, Yoon Jin Lee, Bohyoung Kim, Jin-Hyeok Hwang, Dong Joon Choi

**Affiliations:** 1 Seoul National University Bundang Hospital, Department of Radiology, Seongnam, 13620 Korea; 2 Hankuk University of Foreign Studies, Division of Biomedical Engineering, Yongin, 17035 Korea; 3 Seoul National University Bundang Hospital, Department of Internal Medicine, Seongnam, 13620 Korea

## Abstract

The value of image based texture features as a powerful method to predict prognosis and assist clinical management in cancer patients has been established recently. However, texture analysis using histograms and grey-level co-occurrence matrix in pancreas cancer patients has rarely been reported. We aimed to analyze the association of survival outcomes with texture features in pancreas head cancer patients. Eighty-eight pancreas head cancer patients who underwent preoperative CT images followed by curative resection were included. Texture features using different filter values were obtained. The texture features of average, contrast, correlation, and standard deviation with no filter, and fine to medium filter values as well as the presence of nodal metastasis were significantly different between the recurred (n = 70, 79.5%) and non-recurred group (n = 18, 20.5%). In the multivariate Cox regression analysis, lower standard deviation and contrast and higher correlation with lower average value representing homogenous texture were significantly associated with poorer DFS (disease free survival), along with the presence of lymph node metastasis. Texture parameters from routinely performed pre-operative CT images could be used as an independent imaging tool for predicting the prognosis in pancreas head cancer patients who underwent curative resection.

## Introduction

With a dismal 5-year survival rate of less than 5%, ductal adenocarcinoma of the pancreas remains a lethal disease for most patients^1^. While the only potentially curative treatment proven to prolong survival for pancreas cancer patients is surgical resection, only 15% to 20% of cases are categorized as surgically resectable^2^. In addition, even after curative resection, most pancreatic cancers eventually recur, resulting in a 5-year survival rate for patients who have undergone curative resection of only 25%^3^. Long-term survival after curative resection can be influenced by several factors, including the presence of lymph node metastasis, tumor size, resection margin status, and histologic differentiation^4–8^. However, although adjuvant chemotherapy, which might increase prolonged overall survival, could be considered after curative resection in patients with pathologic risk factors^1,9^, quantitative imaging biomarkers based on preoperative imaging and their associations with clinical outcomes for pancreas head cancer have been rarely documented.

Morphologic heterogeneity is a pathologic finding that is used to characterize a malignant tumor; tumoral heterogeneity indicates the heterogeneous tumor cell population, differentiation, growth pattern, and desmoplastic stroma^10^. The biologic importance of intratumoral heterogeneity in malignant tumors has received attention in recent studies, and there is accumulating evidence that intratumoral heterogeneity at the cellular, molecular, and morphological levels has an important effect on tumor recurrence, therapeutic response, and survival in patients with malignant tumors, including pancreatic cancer^10–12^. From the imaging perspective, intratumoral heterogeneity can be quantified non-invasively by computed tomography (CT) texture analysis, which has a potential role for predicting tumor types, treatment response, and prognosis in various cancers, including head and neck, esophageal, lung, breast, and colorectal cancers^10,13–20^. Given the usefulness of CT texture analysis for prognosis predictions in various cancers, we have hypothesized that the quantitative texture features of pancreas head cancer measured on preoperative CT images might be useful for predicting the clinical outcome of patients with pancreas head cancer after curative resection.

Therefore, the purpose of our study was to evaluate the association of survival outcomes with texture features on preoperative CT images by performing a texture analysis based on a histogram and grey level co-occurrence matrix (GLCM) in patients with pancreas head cancer who have undergone curative resection.

## Results

### Correlation of recurrence with clinical and pathologic features

The clinical and pathologic characteristics of the two groups are listed in Table 1. Out of 88 patients, there were 70 recurrences (79.5%) during the follow-up period. Among the clinical and pathologic variables, only the presence of lymph node metastasis was statistically different between the two groups (43 of 70 [61.4%] vs. 6 of 18 [33.3%], *P* = 0.04). For all the patients, the mean follow-up period was 26.3 months (range, 3.1–89 months) and the mean DFS (disease free survival) was 18 months (range, 0.3–89 months).

**Table 1:** Baseline Patient Characteristics.

Characteristic	Recurrence group (n = 70)	Non-recurrence group (n = 18)	*P* value
Sex			1.000
Male	37	9	
Female	33	9	
Mean age (year)	65.59 ± 10.23	60.44 ± 8.41	0.053
Tumor size			0.574
<2.5 cm	19	6	
≥2.5 cm	51	12	
Lymph node metastasis			0.038
Negative	27	12	
Positive	43	6	
Differentiation			1.000
well or moderately	64	17	
poorly	6	1	
Resection state			0.446
R0	59	17	
R1	11	1	

### Correlation of recurrence with texture features

Regarding the CT texture features without filtration and with the various filter values, the areas under the curve (AUCs) and the optimal cut-off values for diagnosing recurrence determined by ROC curve analysis are summarized in Table 2. Without filtration and with fine (1.0) and medium (1.5 and 2) filter values, the recurrence group showed significantly lower averages, contrast and standard deviations, and higher correlations than the non-recurrence group. Only the average and contrast were significantly different between the two groups with the coarse filter (2.5) value. Applying cross-validation using Leave-one-out cross validation (LOOCV) model yielded the optimal cut-off values in concordance with the previous results (Supplementary Table S1).

**Table 2:** Medial Values of Measured Parameters, AUC and Cut-Off Values on ROC Analyses.

	Recurred group (n = 70)	Non-recurred group (n = 18)	*P* value	AUC	Cut-off value
Filter = 0					
ASM	0.001232	0.001312	0.9505		
Average	1088.4273	1102.1271	0.0021	0.736	≤1098.343478
Standard deviation	16.0277	17.4757	0.0065	0.709	≤16.194633
Kurtosis	0.02222	0.07221	0.6639		
Skewness	0.002466	0.1298	0.3157		
Contrast	192.5875	260.6758	0.00123	0.692	≤204.393377
Correlation	0.00241	0.001702	0.01	0.698	>0.002776
Entropy	6.8646	6.7437	0.7328		
Filter = 1					
ASM	0.0009825	0.0009885	0.6195		
Average	1084.1605	1101.4913	0.0021	0.736	≤1084.931174
Standard deviation	28.0968	30.9342	0.0094	0.699	≤31.434868
Kurtosis	−0.03283	−0.02773	0.5416		
Skewness	−0.04591	0.1201	0.0769		
Contrast	613.7428	856.2486	0.0199	0.679	≤905.806122
Correlation	0.000793	0.000548	0.0127	0.691	>0.000517
Entropy	7.0187	6.9823	0.5835		
Filter = 1.5					
ASM	0.001459	0.001371	0.9464		
Average	1084.307	1101.2969	0.0022	0.735	≤1088.576271
Standard deviation	15.3738	17.2526	0.0151	0.687	≤13.505866
Kurtosis	−0.04924	0.2372	0.1996		
Skewness	0.008841	0.08913	0.4079		
Contrast	111.4899	129.7122	0.0173	0.683	≤89.964225
Correlation	0.003265	0.002783	0.0169	0.683	>0.00398
Entropy	6.6663	6.7282	0.9094		
Filter = 2					
ASM	0.002261	0.002261	0.7999		
Average	1084.9351	1101.203	0.0024	0.733	≤1091.06089
Standard deviation	10.7231	12.3279	0.0314	0.665	≤10.579722
Kurtosis	−0.05305	0.157	0.1689		
Skewness	0.1835	0.1445	0.828		
Contrast	40.7814	46.7342	0.0159	0.685	≤34.290079
Correlation	0.007263	0.005749	0.0386	0.659	>0.008031
Entropy	6.28	6.3254	0.9176		
Filter = 2.5					
ASM	0.002952	0.002879	0.8604		
Average	1085.3756	1101.0749	0.0026	0.731	≤1092.655696
Standard deviation	9.2723	9.7441	0.0769		
Kurtosis	−0.1724	0.2028	0.0859		
Skewness	0.2459	0.1427	0.5835		
Contrast	26.9643	30.6362	0.0314	0.665	≤29.17734
Correlation	0.01012	0.009281	0.0769		
Entropy	6.0562	6.0853	0.9917		

*Note*: ASM = angular second moment; AUC = area under the curve.

### Survival analysis: univariate and multivariate analysis

**Figure 1:**
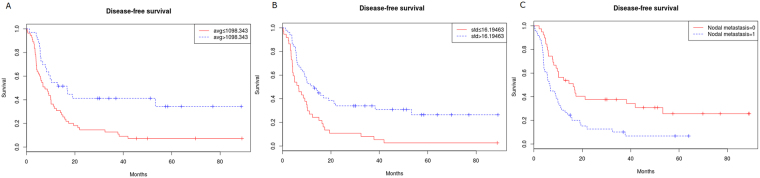
Kaplan-Meier survival curves without filtration show significant difference in disease free survival rates according to stratified (**A**) Average, (**B**) Standard deviation, (**C**) Nodal metastasis with log-rank P values of 0.002, 0.0006 and 0.013, respectively.

**Figure 2:**
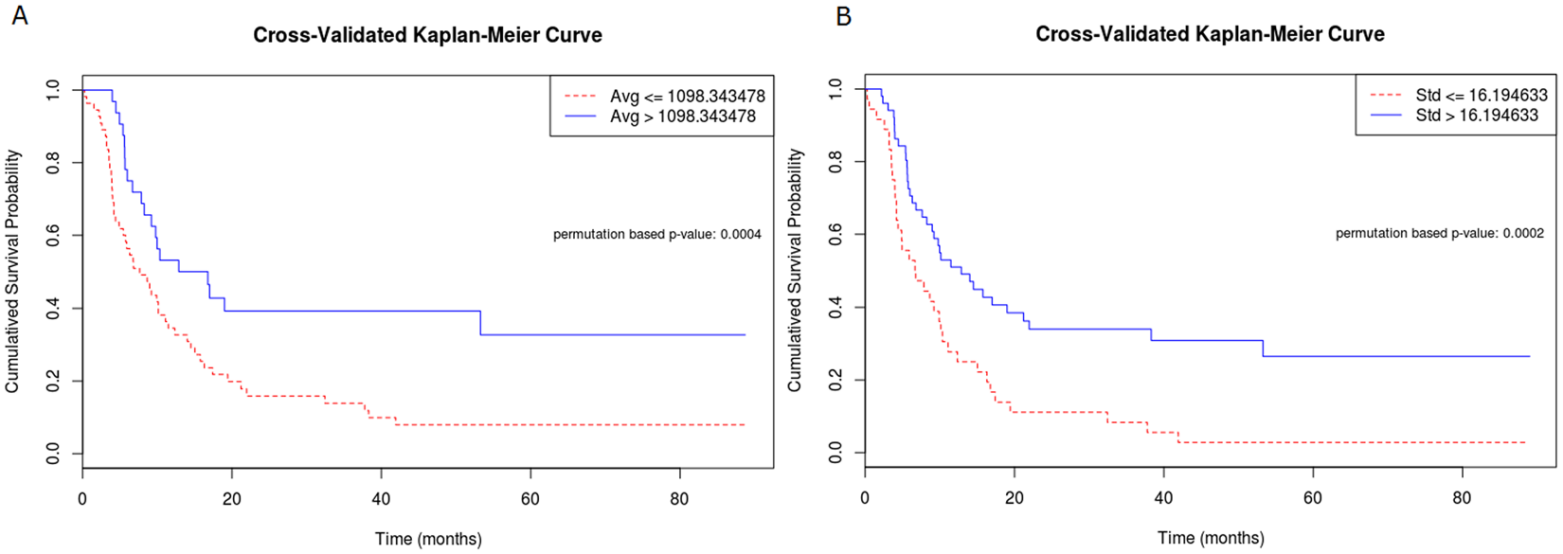
Cross-validated Kaplan-Meier survival curves without filtration show significant difference in disease free survival rates according to stratified (**A**) Average, (**B**) Standard deviation with log-rank P values of 0.0004, 0.0002, respectively.

The results of the univariate Kaplan-Meier analysis and the multivariate Cox proportional hazards model are summarized in Tables 3 and 4, respectively. The univariate Kaplan-Meier analysis with the log-rank test for DFS showed significant differences for the presence of lymph node metastasis, the dichotomized average, contrast, correlation, and standard deviation with no filter and fine to medium filters and for the dichotomized average with the coarse filter (Table 3, Fig. 1). The cross-validation of Kaplan-Meier analysis by LOOVC model for DFS results were in line with the previous results (Table 3, Fig. 2). It showed statistically significant difference for most of the features except for correlation in the filter value of 0, average in the filter value of 1, standard deviation in the filter value of 1.5.

**Table 3:** Kaplan-Meier Survival Analysis for Disease-Free Survival According to Nodal Metastasis and Filter Levels.

	Mean (month)	95% CI for the mean survival	*P* value	*LOOCV *P* value
Nodal status				
pN−	34.259	22.943 to 45.576	0.0013	
pN+	12.959	8.247 to 17.671		
Filter = 0				
Average ≤ 1098.3434781	15.913	9.972 to 21.855	0.002	0.004
Average > 1098.3434781	39.21	25.746 to 52.674		
Contrast ≤ 204.3933771	14.815	8.456 to 21.173	0.0026	0.026
Contrast > 204.3933771	33.434	22.208 to 44.659		
Correlation > 0.002776	13.174	6.723 to 19.624	0.0131	0.12
Correlation ≤ 0.002776	29.473	20.359 to 38.587		
Standard deviation ≤ 16.194633	11.877	6.719 to 17.034	0.0006	0.002
Standard deviation > 16.194633	33.051	22.839 to 43.262		
Filter = 1				
Average ≤ 1084.931174	12.587	7.567 to 17.607	0.0035	0.582
Average > 1084.931174	32.016	21.881 to 42.152		
Contrast ≤ 905.806122	19.058	12.571 to 25.544	0.0083	0.019
Contrast > 905.806122	27.328	18.624 to 36.032		
Correlation > 0.000517	18.89	12.490 to 25.289	0.0056	0.012
Correlation ≤ 0.000517	28.313	19.390 to 37.235		
Standard deviation std ≤ 31.434868	19.003	12.633 to 25.373	0.0169	0.042
Standard deviation std > 31.434868	33.911	20.819 to 47.003		
Filter = 1.5				
Average ≤ 1088.576271	14.087	8.867 to 19.307	0.0061	0.056
Average > 1088.576271	33.331	22.254 to 44.407		
Contrast ≤ 89.964225	9.113	5.053 to 13.174	0.0003	0.001
Contrast > 89.964225	29.913	21.190 to 38.637		
Correlation > 0.00398	9.297	4.777 to 13.817	0.0013	0.258
Correlation ≤ 0.00398	28.51	20.212 to 36.808		
Standard deviation ≤ 13.505866	10.281	5.716 to 14.846	0.0081	0.416
Standard deviation > 13.505866	28.288	19.944 to 36.633		
Filter = 2				
Average ≤ 1091.06089	16.711	10.231 to 23.192	0.0102	0.046
Average > 1091.06089	34.953	22.765 to 47.142		
Contrast ≤ 34.290079	9.909	5.473 to 14.345	0.004	0.062
Contrast > 34.290079	28.685	20.252 to 37.118		
Correlation > 0.008031	13.208	7.956 to 18.461	0.0166	0.049
Correlation ≤ 0.008031	29.884	20.293 to 39.475		
Standard deviation ≤ 10.579722	12.884	8.028 to 17.740	0.0085	0.009
Standard deviation > 10.579722	31.134	21.078 to 41.190		
Filter = 2.5				
Average ≤ 1092.655696	16.533	10.171 to 22.896	0.0075	0.042
Average > 1092.655696	35.692	23.260 to 48.124		

*Note*: *LOOCV (Leave-one-out cross validation).

**Table 4:** Multivariate Cox Survival Analysis of Variables for Disease-Free Survival.

	HR	95% CI of HR	*P* value
Filter = 0			
Nodal metastasis	2.0375	1.2441 to 3.3378	0.0047
Average	0.5599	0.3201 to 0.9791	0.042
Standard deviation	0.5745	0.3467 to 0.9521	0.0315
Filter = 1			
Nodal metastasis	2.1257	1.2988 to 3.4793	0.0027
Average	0.5532	0.3254 to 0.9406	0.0288
Correlation	1.9806	1.0785 to 3.6364	0.0275
Filter = 1.5			
Nodal metastasis	1.957	1.1917 to 3.2137	0.008
Contrast	0.4665	0.2822 to 0.7712	0.003
Filter = 2			
Nodal metastasis	2.1457	1.3117 to 3.5099	0.0024
Standard deviation	0.5540	0.3459 to 0.8874	0.014
Filter = 2.5			
Nodal metastasis	2.1814	1.3344 to 3.5660	0.0019
Average	0.5190	0.3161 to 0.8521	0.0095

*Note*: HR = hazard ratio.

In the multivariate Cox proportional hazards model, the presence of lymph node metastasis was an independent factor that showed a significant association with DFS regardless of the applied filter (hazard ratio [HR], 1.957 to 2.181). Various texture features—including average filter values of 0, 1, and 2.5; standard deviation in the filter values of 0 and 2; contrast in the 1.5 filter value; and correlation in the filter value of 1—served as independent prognostic factors for predicting poorer DFS (Table 4). Overall, homogeneous texture features (lower standard deviation and contrast and higher correlation) with a lower average value from the texture analyses were significantly associated with poorer DFS.

## Discussion

In this study, we have demonstrated the prognostic value of texture features of preoperative CT images using histograms and GLCM analyses in patients with pancreatic head cancer who have undergone curative resection. Our results show that lower average values with homogeneous features (lower standard deviation and contrast and higher correlation), along with the presence of lymph node metastasis, are significantly associated with poorer DFS, although the *P* values and HRs varied according to the applied filters.

In our study population, lower average values (without filtration and with filters 1.0 and 2.5) of pancreatic head cancer were found to be negative prognostic factors for DFS after curative resection in a multivariate Cox proportional hazards model. As the pixel histogram average represents the brightness or mean gray-level intensity of a region, a lower average on the texture analysis indicated a lesion with low attenuation. One of characteristic pathologic features of pancreas cancer is the presence of intense fibrosis in the tumor, which is known as desmoplastic reaction^21^. Previous studies have demonstrated that scirrhous carcinomas with abundant fibrosis and relative sparse tumor cells in the stomach, bile duct, breast, and colon have a poor prognosis^22–25^. Now the evidence is accumulating that the fibrous component of a tumor correlates with its malignant behavior and contribute to therapeutic resistance^26^. Although the significance of the fibrotic component of pancreas cancer is still unclear, peritumoral fibroblasts in pancreatic cancer have been shown to overexpress SPARC (secreted protein acidic and rich in cysteine), which is a marker of poor prognosis when expressed in the stroma^27^. Furthermore, a desmoplastic reaction in pancreas cancer is thought to be responsible for metastasis, as well as chemotherapy resistance, by reducing the amount of drug delivered to the tumor^28^. Because pancreatic cancer frequently has an abundant fibrotic stroma, which is seen as a hypo-attenuating mass in the early arterial phase with progressive delayed enhancement^29,30^, we believe that the lower average observed on the pancreas phase images reflects pancreatic cancer with abundant desmoplastic reactions. Other studies have suggested that iso-attenuating pancreatic cancers on early-phase images tend to display less desmoplastic change within the mass and show better survival outcome^25,31–36^. Studies involving diffusion MRI have concluded that the degree of fibrosis in pancreatic cancer cases correlates with diffusion restrictions related to poor prognosis^37,38^ and that it could be used to monitor treatment response^39^. We speculate that pancreatic head cancer with a lower average may reflect an imaging phenotype of pancreatic cancer with abundant desmoplastic reactions that represents an aggressive subset of this cancer and that it might be related to poorer survival outcomes.

Our results are in line with the findings of Cassinotto *et al*.^40^, who demonstrated that hypo-attenuating pancreatic cancer in the portal-venous phase on CT scans showed shorter DFS. However, the contrast between normal parenchyma and pancreatic cancer is greater in the pancreatic phase than in the portal-venous phase, and tumors normally demonstrate peripheral enhancement of the tumor in the portal-venous phase^41–43^. Therefore, our data obtained from the pancreatic phase would be better for representing the entire tumor mass as well as the internal heterogeneity compared to the data from the portal-venous phase.

Also, interestingly, our study has revealed that both first- (a lower standard deviation without filtration and with the 2.0 filter) and second-order statistics (a lower contrast with the 1.5 filter and a higher correlation with the 1 filter) representing intratumoral homogeneity are related to poorer DFS in the multivariate Cox proportional hazards model. The first-order statistics, calculated from a histogram of pixel values, were based on the gray-level frequency distribution and represent a single pixel value rather than its spatial relation to adjacent pixels^13,44^. Instead, secondary parameters, calculated using GLCM, show the spatial relationship between one pixel and another. These secondary parameters have the advantage of being able to quantify the overall texture content^13,45^. Our study differs from the work of Cassinotto *et al*.^40^, who only used first-order statistics to perform a texture analysis in pancreatic cancer, in that our results that were obtained using both first-order and second-order texture measures to better quantify heterogeneity within the pancreatic tumors. Our results suggest that homogenous features are correlated with poorer survival outcomes, in contrast to the majority of previous studies that found that increased tumoral heterogeneity on CT images is related to poorer clinical outcomes^17,46–48^. Heterogeneity is a well-recognized feature of malignant tumors and presumably reflects alterations in the tissue microenvironment due to cell infiltration, angiogenesis, necrosis, and myxoid changes^13,48,49^. In prior studies, tumor heterogeneity measured on CT images correlated with histologic findings of an irregular, disorganized architectural distortion from angiogenesis and hypoxia in primary colorectal cancer and non-small-cell lung cancer^50,51^. However, contradictory findings were found in studies of primary^15^ and metastatic colorectal cancer^52^, where texture variables representing less heterogeneity (e.g., lower entropy and standard deviations) were associated with poorer survival. Based on our study results, as well as those of the studies mentioned above (15, 53), we conjecture that homogeneous texture features could represent more aggressive behavior in tumors, thereby representing higher cellular density or dense desmoplasia. Our study results therefore imply that texture analysis on pre-operative CT scans may be potentially used to identify patients who have a higher chance of recurrence after curative resection and therefore would benefit from extensive postoperative surveillance and adjuvant therapy. Moreover, multiple ongoing studies are focused on validating the benefit of neoadjuvant chemotherapy in patients with resectable or borderline resectable pancreatic cancer, although there are no data that clearly suggest improved survival with neoadjuvant chemotherapy^53^. In addition to endoscopic ultrasound or measuring serum CA 19-9 levels for the selection of candidates for neoadjuvant therapy^54–56^, the ability to stratify prognosis in patients with initially resectable pancreas head cancer by performing texture analyses of routine preoperative CT images could be helpful for selecting candidates for neoadjuvant chemotherapy. Further research is warranted to confirm the correlation between texture features and clinical outcomes in a prospective, larger cohort and to determine whether the prognostic information from texture analyses could be clinically utilized for patients with pancreatic head cancer.

Several limitations need to be addressed with respect to our study. First, as this study was retrospectively designed, the possibility of selection bias should be considered. Second, we did not take into account potential variables affecting tumor enhancement on the contrast-enhanced CT scans, including cardiac output, body mass, and blood volume. Third, although the texture parameters are relatively insensitive to the CT acquisition factors^57^, the use of three different types of scanners in our study might have resulted in the inherent variability of the texture features. Future studies using the same scanner and CT acquisition protocol to reduce other possible factors affecting texture analysis are required. Fourth, given that the external validation was not performed in our study, we cannot be certain that the result in our study could be applied to the external, prospectively recruited patients. Nonetheless, the LOOCV used for cross-validation was shown to strengthen the reliability of our study results. Thus, while the results of our study cannot be immediately applied to clinical practice, further prospective validation studies using large multicentre datasets are warranted. Lastly, contrary to several reports suggesting that 3-dimensional (3D) analysis would better account for tumor heterogeneity^58^, we performed a 2-dimensional (2D) quantitative tumor analysis by selecting the single axial image with largest tumor area. In addition, aside from the fact that 3D whole-tumor analysis is complex and time-consuming, recent studies have shown that there is no difference between 2D and 3D tumor analyses^52^.

Despite several limitations in our study, it is the first to investigate the association of first and second texture features with the prognosis in pancreas cancer head patients. In the era of Radiomics, the need for standardization is increasing to provide clinically relevant results. The number of patients included in our study was within the suggested value (10–15 patients per feature) to test prognostic power of texture features. Furthermore, we have provided details of methods used in the analysis and included clinically important variables in the analysis. Our study provides that texture-feature-based image analysis holds promise in predicting prognosis in pancreas head cancer patients, and that the prospective clinical studies may be needed to better delineate the potential of this approach.

In conclusion, lower average and standard deviation values from CT texture analyses are associated with poorer survival outcomes in pancreas head cancer patients who underwent curative resection. Texture analysis features from routinely performed pre-operative CT images could be used as an independent imaging parameter for predicting the prognosis in these patients.

## Materials and Methods

Seoul National University Bundang Hospital institutional review board approval was obtained for this study, and informed consent was waived. All methods were performed in accordance with the relevant guidelines and regulations.

### Patients

**Figure 3:**
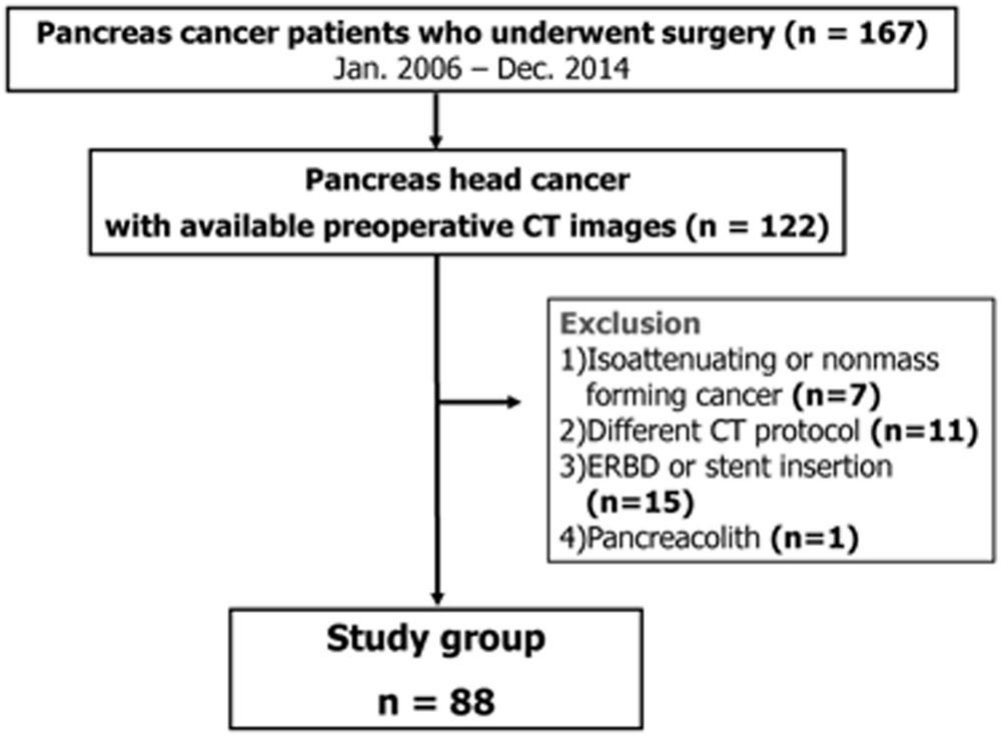
Flow chart showing patient selection criteria of our study.

From January 2006 to December 2014, 167 patients underwent resection for pancreas cancer in our institution. Among them, 122 patients who had a histopathologic diagnosis of ductal adenocarcinoma in the pancreas head were initially included in this study. Of these 122 patients, 27 patients were excluded from this study for the following reasons, as these factors could potentially influence the texture values: biliary stent placement along the common duct prior to CT examination (n = 15), different CT protocols (n = 11), and pancreatolith in the pancreas head area (n = 1). Additionally, 7 patients were excluded because their pancreas head cancers were not identifiable on the initial CT images. Finally, 88 patients were included as the sample group for our study (Fig. 3). None of these included 88 patients had undergone either preoperative radiation or chemotherapy.

### CT imaging protocol

All patients underwent preoperative contrast-enhanced CT imaging with a pancreas protocol. After the acquisition of non-contrast images, iopromide, an intravenous contrast material (Ultravist 370; Bayer, Berlin, Germany) was injected via the antecubital vein using a power injector (Stellant D; Medrad, Indianola, PA) at a dose of 2 mL per kilogram of body weight at a rate of 3 mL/sec. CT scans of the pancreatic and portal venous phase were initiated after the bolus contrast media injection with delays of 20 and 60 seconds after aortic enhancement of 150 HU, respectively. Non-contrast and pancreatic phase images were acquired from the diaphragm to the umbilicus level, and portal venous phase images were obtained from the diaphragm to symphysis pubis level. Images were acquired with 16- (n = 35), 64- (n = 39), or 128- (n = 14) multi-detector CT scanners (Mx 8000, Brilliance 64, iCT256; Philips Medical Systems, Cleveland, OH). The scanning parameters were as follows: 16 × 1.5, 64 × 0.625, or 128 × 0.625 mm collimation; a rotation speed of 0.5 s; a pitch of 1.25, 0.641, or 0.993; a kvP of 120. Effective mAs ranged from 72 to 385 mAs using an automatic tube current modulation technique (Dose-Right; Philips Medical Systems). The CT images were reconstructed using filtered back projection with 4-mm thick sections at 3-mm increments.

### Quantitative texture analysis

**Figure 4:**
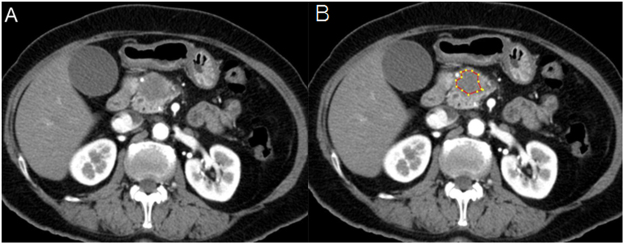
An example of quantitative texture analysis of the pancreas head cancer. (**A**) Axial pancreatic phase CT scan shows a low attenuating mass in the pancreas head. (**B**) ROI was drawn excluding fat or air densities for texture analysis.

The pancreatic-phase CT images were retrieved from the picture archiving and communication system and transferred to an independent workstation for further texture analysis using software built in-house. After selecting the single axial pancreatic-phase CT image^41^ showing the largest cross-sectional area of the pancreas head cancer, a polygonal region of interest (ROI) was manually drawn as large as possible within the tumor border with the consensus of two radiologists (K.Y.H. and Y.G.B., with 20 and 3 years of experience in abdominal imaging, respectively) who were blinded to the pathologic and clinical outcomes (Fig. 4). Particular attention was paid to avoiding the peripancreatic vessels while delineating the ROIs for each case. Areas of air and fatty tissues were removed from the analyses by excluding any pixels with attenuation values less than 0 Hounsfield units. Although the contouring was performed on the pancreatic-phase CT images, the portal-venous-phase CT or magnetic resonance imaging (MRI) scans were also reviewed to check whether the ROIs were accurately drawn. The median tumor areas and the number of pixels in the ROIs for the texture analyses were 132.8 cm^2^ (range, 61.3 to 597.6) and 433.3 (range, 164 to 1,685), respectively.

Laplacian of the Gaussian band-pass filter was applied to detect intensity changes within the images smoothened by Gaussian distribution based on the filter sigma value^17,59^. This resulted in the images displaying features at different scales (from fine to coarse textures) associated with filter sigma values within the ROI around the pancreas head cancer. The scale was determined by filter sigma values of 1.0 (fine texture, filter width 4 pixels), 1.5 to 2.0 (medium texture, filter width 6–10 pixels), and 2.5 (coarse texture, filter width 12 pixels)^17^. The degree of image smoothening was proportional to the filter value: a higher (or lower) filter value enabled the extraction of a coarse (or fine) texture by smoothening the images to a greater (or lesser) degree^59^. The distributions of pixel values of the gray-level histograms within the ROIs were characterized by average (mean intensity of the gray-level distribution), standard deviation (the degree of dispersion), kurtosis (flatness of the histogram), and skewness (asymmetry of the histogram). Texture parameters, including angular second moment (or energy; uniformity in gray-level distribution), entropy (randomness of pixel distribution), correlation (measurement of gray-level linear dependence), and contrast (measurement of local variations) were calculated by GLCM, which represents the spatial dependence relationship between groups of neighboring pixel intensity values^13,14,45^. In general, a higher standard deviation of the pixel distribution, a higher kurtosis, a positive or negative skewness, a higher entropy, and a higher contrast and lower angular second moment and correlation represented increased heterogeneity^14–17,45^.

### Review of pathologic and clinical follow-up data

The pathologic and clinical follow-up data were reviewed by one radiologist (Y.J.L., with 9 years of experience in abdominal imaging). The final histopathologic reports of the surgically excised specimens were also reviewed for tumor size, presence of lymph node metastasis, resection margin involvement, and pathologic differentiation according to the 7^th^ American Joint Committee on Cancer staging system^60^. The pathologic results were dichotomized as follows: smaller than 2.5 cm or larger than or equal to 2.5 cm for size, positive or negative for lymph node metastasis, positive (R1) or negative (R0) for surgical margins, and well to moderately or poorly differentiated pathologic differentiation^61^. After surgery, all patients underwent clinical follow-up according to our institutional protocol, including serum cancer antigen (CA) 19-9 measurement and CT examinations at 3- to 6-month intervals. Medical records and CT examinations following surgical resection were reviewed, focusing on the presence and date of tumor recurrence or death and last follow-up date. Tumor recurrence was determined by the presence of locoregional recurrence or distant metastasis documented on a patient’s medical record based on physical examination, laboratory findings, follow-up imaging studies, and pathologic reports of biopsy samples, if available. Then the patients were classified into recurrence and non-recurrence groups. DFS was defined as the period from resection to the diagnosis of the tumor recurrence or to any cause of death. The final data were collected on March 31, 2017. Patients without recurrence on the date of the most recent follow-up were censored in the analysis.

### Statistical analysis

The clinicopathologic results and CT texture features were compared between the recurrence and non-recurrence groups. The univariate analysis for categorical variables was performed using the chi-square test. A Mann-Whitney U test was performed to compare the continuous variables between the two groups. To dichotomize the texture features with or without filters for the survival analysis, the optimal cut-off values were determined by the value which maximizes the sum of sensitivity and specificity on a receiver-operating characteristic (ROC) curve analysis. To improve the power of prediction, additional cross-validation of the results using Leave-one-out cross validation (LOOCV) test was adopted. In LOOCV, multiple rounds of ROC analysis are carried out by using the training data and then the validation data are assigned to dichotomized group based on the cut-off point. The cut-off point selected most frequently was defined as optimized cut-off value in the LOOVC analysis. DFS was analyzed by using Kaplan-Meier method based on each of the cut-off values calculated by the ROC curve analysis, and comparisons of the dichotomized variables between groups were performed by a log-rank test. Additionally, LOOCV cross-validated Kaplan-Meier analysis was performed (Table 3). A multivariate Cox proportional hazards model with a hierarchical forward step-wise procedure was used to assess whether the texture features with or without filters were independently and significantly associated with DFS. Variables with *P* values less than 0.05 in the univariate Kaplan-Meier analysis were entered into a multivariate Cox proportional hazards model. All analyses were performed using SPSS version 14.0 (Chicago, IL) and Medcalc version 12.1.4.0 (Medcalc Software, Ostend, Belgium). *P* values less than 0.05 were considered to be statistically significant.

## Electronic supplementary material

Supplementary table 1
