# Supplementary material for: Tumor heterogeneity of pancreas head cancer assessed by CT texture analysis: association with survival outcomes after curative resection
Source: Sci Rep. 2018 May 8;8:7226. doi: 10.1038/s41598-018-25627-x (PMC5940761; doi:10.1038/s41598-018-25627-x)
Supplement: Supplementary file 1 — Supplementary table 1 [file 41598_2018_25627_MOESM1_ESM.pdf]

Tumor heterogeneity of pancreas head cancer assessed by CT texture analysis: association with survival outcomes after curative resection

Gabin Yun, MD[1], Young Hoon Kim, MD[1,*], Yoon Jin Lee, MD[1] , Bohyoung Kim, PhD[1,2], Jin-Hyeok Hwang, MD, PhD[3], Dong Joon Choi[1]

Supplementary Table S1. LOOCV optimal cutoff and Mean AUC

	Filter = 0	Filter = 1.0	Filter = 1.5	Filter = 2.0	Filter = 2.5
Average	≤1098.343 (0.736)	≤1084.931 (0.736)	≤1088.576 (0.735)	≤1091.061 (0.733)	≤1092.656 (0.731)
Standard Deviation	≤16.19463 (0.709)	≤31.43487 (0.699)	≤13.50587 (0.687)	≤10.57972 (0.665)	
Contrast	≤204.3934 (0.692)	≤905.8061 (0.679)	≤89.96422 (0.683)	≤34.29008 (0.685)	≤29.17734 (0.665)
Correlation	>0.002776 (0.698)	>0.000517 (0.691)	>0.00398 (0.683)	>0.008031 (0.659)	

Note—AUC values are in parentheses
